# Anticancer activities of natural antimicrobial peptides from animals

**DOI:** 10.3389/fmicb.2023.1321386

**Published:** 2024-01-17

**Authors:** Baozhen Qu, Jiangshui Yuan, Xueli Liu, Shicui Zhang, Xuezhen Ma, Linlin Lu

**Affiliations:** ^1^Qingdao Cancer Prevention and Treatment Research Institute, Qingdao Central Hospital, University of Health and Rehabilitation Sciences (Qingdao Central Medical Group), Qingdao, China; ^2^Department of Clinical Laboratory, Qingdao Hospital, University of Health and Rehabilitation Sciences (Qingdao Municipal Hospital), Qingdao, China; ^3^Medical Ethics Committee Office, Qingdao Central Hospital, University of Health and Rehabilitation Sciences (Qingdao Central Medical Group), Qingdao, China; ^4^College of Life and Geographic Sciences, Key Laboratory of Biological Resources and Ecology of Pamirs Plateau in Xinjiang Uygur Autonomous Region, Kashi University, Kashi, China; ^5^Institute of Evolution & Marine Biodiversity, Ocean University of China, Qingdao, China; ^6^Department of Oncology, Qingdao Central Hospital, University of Health and Rehabilitation Sciences (Qingdao Central Medical Group), Qingdao, China

**Keywords:** antimicrobial peptides, anticancer peptides, animals, marine, terrestrial, mechanisms, clinical applications

## Abstract

Cancer is the most common cause of human death worldwide, posing a serious threat to human health and having a negative impact on the economy. In the past few decades, significant progress has been made in anticancer therapies, but traditional anticancer therapies, including radiation therapy, surgery, chemotherapy, molecular targeted therapy, immunotherapy and antibody-drug conjugates (ADCs), have serious side effects, low specificity, and the emergence of drug resistance. Therefore, there is an urgent need to develop new treatment methods to improve efficacy and reduce side effects. Antimicrobial peptides (AMPs) exist in the innate immune system of various organisms. As the most promising alternatives to traditional drugs for treating cancers, some AMPs also have been proven to possess anticancer activities, which are defined as anticancer peptides (ACPs). These peptides have the advantages of being able to specifically target cancer cells and have less toxicity to normal tissues. More and more studies have found that marine and terrestrial animals contain a large amount of ACPs. In this article, we introduced the animal derived AMPs with anti-cancer activity, and summarized the types of tumor cells inhibited by ACPs, the mechanisms by which they exert anti-tumor effects and clinical applications of ACPs.

## 1 Introduction

Cancer has emerged as a major cause of human death worldwide, which poses a serious threat to public health and have a negative impact on the economy (Deo et al., 2022). In the past few decades, our understanding of the origin of cancer has developed to a widespread acceptance that cancer is the result of an evolutionary process driven by natural selection of mutations, subsequent genetic and carcinogenic mutations (Somarelli et al., 2020). Based on statistics from the World Health Organization (WHO) in 2019, cancer is the first or second major cause of death before the age of 70 in 112 out of 183 countries, and ranks third or fourth out of 23 other countries (Sung et al., 2021). According to GLOBOCAN 2020 published by the International Agency for Research on Cancer (IARC), in 2020, there were 19.3 million new cancer cases and 9.9 million new cancer deaths worldwide (Ferlay et al., 2020). The number of cancer deaths will keep growing, with an estimated 13 million deaths by 2030 (Asasira et al., 2022). Among all the cancers, female breast cancer is the most commonly diagnosed cancer, closely followed by lung, colorectal, prostate, and gastric cancers. Lung cancer is the leading cause of cancer death, followed by colorectal, liver, gastric and female breast cancers (Ferlay et al., 2020). The characteristics of all types of neoplastic cells are unregulated cell growth caused by genetic mutations in a small amount of inherited or environmental stimuli, which can achieve replication immortality, avoid cell death, escape from the host immune system, and so on (Schiliro and Firestein, 2021). Well-known signaling pathways, such as the mitogen-activated protein kinase (MAPK), nuclear factor kappa-B (NF-κB), Wnt, transforming growth factor-β (TGF-β), Notch, Hippo, Janus kinase (JAK)/signal transducer and activator of transcription (STAT), Hedgehog (Hh), and phosphoinositide 3-kinase (PI3K)/AKT pathways, as well as transcription factors, including heat shock transcription factor 1 (HSF1), hypoxia-inducible factor (HIF), Snail, P53, and Twist, constitute complex regulatory networks to modulate the formation, activation, heterogeneity, metabolic characteristics and malignant phenotype of cancers (Fang et al., 2023).

The current therapeutic strategies for treating cancer, include radiation therapy, surgery, chemotherapy, molecular targeted therapy, immunotherapy and antibody-drug conjugate (ADC). For example, conventional chemotherapy, one of the most widely used cancer treatment methods, kills cancer cells by inhibiting cell growth or division. Chemotherapeutics (i.e., alkylating agents, antimetabolic agents, antineoplastic antibiotics, platinating agents, and plant-derived alkaloids) exert anti-tumor effects through direct cytotoxic effects or indirectly by affecting the cell cycle (Okunaka et al., 2021). Progress in molecular targeted drugs has prolonged survival (Li et al., 2023), particularly in patients with lung cancer harboring epidermal growth factor receptor (EGFR) mutations (i.e., erlotinib, gefitinib, afatinib, dacomitinib, and osimertinib) or anaplastic lymphoma kinase (ALK) rearrangement (i.e., ceritinib, alectinib, brigatinib, ensartinib, lorlatinib), and in patients with breast cancer harboring human epidermal growth factor receptor 2 (HER2) amplification (i.e., trastuzumab). Furthermore, cancer immunotherapies targeting the interaction of programmed cell death 1 (PD-1) with its major ligands, PD-L1 and PD-L2 are considered to usher in the era of modern oncology. Drugs that block PD-1 (pembrolizumab, nivolumab, and cemiplimab) or PD-L1 (atezolizumab, durvalumab, and avelumab) promote endogenous anti-tumor immunity and have been considered effective strategies for cancer treatment due to their broad activity spectrum (Topalian et al., 2020). It is worth noting that in recent years, ADC has become a promising cancer treatment method for solid and hematological malignancies. ADC assembles cytotoxic drugs (payloads) by covalently linking them to monoclonal antibodies (mAbs) and delivering them to tumor tissues expressing their specific antigens, with the advantage of low toxicity and improved treatment rates (Fuentes-Antrás et al., 2023). Currently, the US Food and Drug Administration (USFDA) has approved 13 types of ADCs for the treatment of various types of solid tumors and hematological malignancies (Maiti et al., 2023). However, there are still several obstacles that will affect and limit their curative effect. These treatment modalities have been found to have no specificity for cancer cells resulting in affecting the cell division of normal cells, thereby damaging the repair of healthy tissues (Ziaja et al., 2020). Besides, cancer patients often develop multi-drug resistance (MDR) to chemotherapeutics acquired by tumors (Ziaja et al., 2020). Thus, the development of novel tumor-targeted therapies that could effectively target tumor cells with lower/minor toxicity to normal cells and battle drug resistance is a top priority for treating cancer. In recent decades, researchers have focused on finding new anticancer strategies and have shifted their attention to antimicrobial peptides (AMPs).

AMPs are natural bioactive peptides with diverse structural properties that are produced by bacteria, fungi, plants and animals, and act as the first line of defense against invading pathogens (Wang G. et al., 2022). In 1981, the first important animal derived AMP named cecropins was identified from the Cecropia silk moth, followed by magainin from *Xenopus laevis* in 1987 (Steiner et al., 1981; Zasloff, 1987). After that, numerous other natural AMPs were discovered in almost all organisms (Han et al., 2021; Ma et al., 2021). Since then, more than 3000 AMPs have been deposited in the Antimicrobial Peptide Database.^1^ In addition to having broad-spectrum antibacterial effects, an increasing number of AMPs have been proven to have other physiological functions including antifungal, antiviral, antioxidative, antithrombotic, antihypertensive and immunomodulatory (Macedo et al., 2021; Quah et al., 2023). Importantly, many AMPs also demonstrated possess antitumor activity. Most AMPs rely on destructing cell membranes or changing cell membrane permeability to kill bacteria or cancer cells (Lyu et al., 2019), but some AMPs can also via non-membranes disruptive mechanisms exert anti-cancer effects. For instance, cecropins and LL-37 could induce apoptosis and necrosis of tumor cells (Qin et al., 2019; Yang et al., 2021), melittin could suppress tumor angiogenesis and reactivate immune cells (Zhang and Chen, 2017; Duffy et al., 2020).

Anticancer peptides (ACPs) have many unique advantages compared to chemotherapy drugs, such as biocompatibility, efficient therapeutic efficacy, low risk of drug resistance appearing in tumor cells, and limited or no toxicity against mammalian cells (Karami Fath et al., 2022; Lath et al., 2023). In addition, ACPs have immunogenicity and low difficulty in synthesis and modification, with a short half-life *in vivo*, making it possible for them to be put into clinical anti-cancer drug candidates (Karami Fath et al., 2022). In this review, we summarize and describe the anticancer effects and the mode of action of AMPs derived from marine and terrestrial animal sources based on their order of evolution, and we further discuss the clinical applications of ACPs.

## 2 The structural classifications and selective recognition to tumor cells of AMPs

Generally, the naturally produced AMPs are cationic and amphipathic, with the net charge at neutral pH ranging from + 2 to + 9 (Costa et al., 2011). Most AMPs are short in length, usually containing 10–50 L-amino acids, which are rich in lysine, arginine and large amounts of other hydrophobic residues (>30%) (Maroti et al., 2011). So why can AMPs inhibit or kill bacteria? Notably, the surfaces of Gram-positive and Gram-negative bacteria are rich in lipopolysaccharides and teichoic acid, which result in a net negative charge generated on the cell membrane surface. Therefore, the cationic and amphipathic nature of AMPs can generate electrostatic interactions with negative charges on the surface of bacteria and interact with various hydrophilic and hydrophobic components (Luo and Song, 2021). At present, the AMPs can be classified as α-helical, β-sheet, mixed α-helical/β-sheet, as well as cyclic and unstructured (neither α-helix nor β-sheet) AMPs according to the secondary structure (Liang et al., 2020). Among them, the α-helix peptides are the most studied type of AMPs. In aqueous solution, α-helical AMPs possess a linear structure, but when come in contact with bacterial membranes or organic solvents these adopt an amphipathic helical structure, which contributes to insert into the cell membrane (Mahlapuu et al., 2016). Specifically, α-helical AMPs adhere to negatively charged bacterial membranes through electrostatic interactions, inserting their hydrophobic domains into the bacterial membrane, resulting in membrane deformation (Liang et al., 2020). The two most studied and representative peptides in this group are human LL-37 and magainin 2 (MG2) (see sections “3.2.2 ACPs derived from amphibians and 3.2.4 ACPs derived from mammals” for details). β-sheet AMPs are typically molecules composed of at least two antiparallel β-sheet, which contain 6 to 8 cysteine residues and are further stabilized by forming two or more disulfide bonds (Lewies et al., 2015). At present, defensins are the most widely studied family of β-sheet AMPs (see section “3.2.4 ACPs derived from mammals” for details). Tachyplesin I is another β-sheet AMPs which forms a uniquely rigid and stable β-hairpin with three tandem tetrapeptide repeats (Luan et al., 2021). The third type, α-helix/β-sheet mixed structure, stabilized three to four disulfide bond bridges, is another major structural motif of some AMPs. For instance, cysteine-stabilized αβ (CSαβ) defensins consist of a single α-helix and β-sheets with two or three antiparallel chains, with the length varying from 34 to 54 amino acid residues (Dias Rde and Franco, 2015). Insect heliomicin and fungi defensin eurocin are two typical representatives of CSαβ defensins. The fourth type of cyclic peptides contains one or two disulfide bonds to form this conformation. The typical representative is bacteriocins, which normally come from bacteria rather than animals. For example, plantacyclin B21AG and bacteriocins enterocin 7A are two circular bacteriocins, which display antimicrobial activity. Finally, the unstructured (neither α-helix nor β-sheet) AMPs rich in specific amino acids such as tryptophan, proline, and arginine, and typically around 15 amino acids, exhibit a linear structure (Schibli et al., 2002). This unstructured type of AMPs usually presents a disordered or loose curly structure before interaction with the lipid bilayer, and will quickly fold into active conformation once contact with the membrane (Johansson et al., 1998). Indolicidin, from cytoplasmic granules of bovine neutrophils, is a typical linear AMP rich in tryptophan and proline (Smirnova et al., 2020). LF11 is another short linear AMP based on the human lactoferrin (Zhang et al., 2022).

In the manner of cell membrane destruction modes, ACPs and AMPs have similar mechanisms leading to thinning, forming pores, changing the degree of curvature of the cell membrane, etc. These actions can lead to changes in the overall membrane potential of the cell membrane, and loss of membrane potential gradient, thus stopping the synthesis of ATP and the termination of cell metabolism, and ultimately leading to the death of cells (Chiangjong et al., 2020; Luan et al., 2021). There are three commonly recognized types of cell membrane destruction modes, i.e., the barrel-stave model, the toroidal model, and the carpet model (Li et al., 2021; Erdem Büyükkiraz and Kesmen, 2022). The barrel-stave model has the most extensively study. In this model, after binding to the cell membrane, ACPs/AMPs are arranged vertically with the cell membrane and aggregate with each other to form a cylindrical insertion into the cell membrane, thereby forming pores on the cell membrane. In the toroidal model, after the ACPs/AMPs combine with the cell membrane, the cell membrane bends inward to form a hole, which is composed of the hydrophilic head of the lipid bilayer and ACPs/AMPs. The carpet model has a strong destructive effect on cell membranes. In the model, ACPs/AMPs bind to the cell membrane and are parallel to the surface of the cell membrane. After reaching a certain concentration of ACPs/AMPs, the cell membrane is broken down into small particles one by one.

In the manner of non-membrane destruction modes, ACPs could display anti-tumor activity by mediating the necrosis or apoptosis of cancer cells, inhibiting angiogenesis, immune cell recruitment and activating certain regulatory functional proteins (Huan et al., 2020; Jafari et al., 2022).

Why do ACPs recognize tumor cells specifically? In this respect, the abundant presence of anions on the surface of tumor cells significantly increases the electrostatic interaction between ACPs and the surface of cancer cells. However, compared with tumor cells, healthy cell membranes have zwitterion phosphatidylcholine and sphingomyelin in an outer leaflet and anionic phosphatidylserine (PS) and the phosphatidylethanolamine in the inner leaflet with the asymmetric distribution (Chiangjong et al., 2020). The outer leaflet of the plasma membrane of tumor cells usually contains a large amount of negatively charged PS, which is 3–7 times more than normal cells (Haider and Soni, 2022). In addition, anionic molecules such as O-glycosylated mucins, sialylated gangliosides and heparin sulfate, which are overexpressed in tumor tissue and closely related to the occurrence and progression of tumors, also greatly increase the number of negative charges on the surface of tumor cells (Luong et al., 2020). Moreover, the differences in membrane fluidity and cell surface area between tumor cells and normal cells are also considered important reasons for the preferential action of ACPs on tumor cells. Cholesterol accumulates in a larger amount on the cell membrane of normal cells than in cancer cells, which could effectively reduce the membrane fluidity to prevent cationic peptides from inserting into the cell membrane (Norouzi et al., 2022). On the contrary, most cancer cells have lower cholesterol content on their membranes, making them more fluid than normal cells (Luan et al., 2021), leading to ACP being more prone to disrupting membrane stability. Another interesting characteristic of cancer cells is the expression of a large number of microvilli in their plasma membrane, which increases the surface area of the membrane and thus increases the affinity of ACPs to tumor cells (Luan et al., 2021).

## 3 Animal sources of AMPs with anticancer activity

**FIGURE 1:**
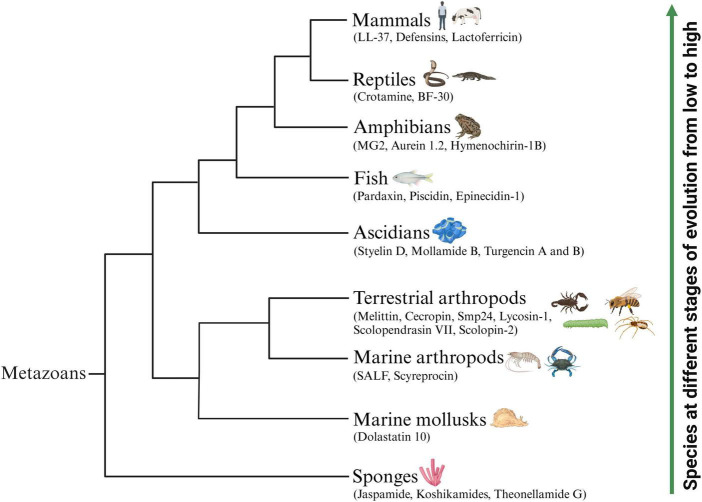
Schematic representation of representative animal-origin ACPs according to the animal’s evolution order. Some known ACPs which have been identified from various marine animals mainly concentrated in sponges, mollusks, marine arthropods, ascidians and fish. Several representative ACPs also isolated from terrestrial animals including terrestrial arthropods, amphibians, reptiles and mammals.

The natural environment is considered a vital source of novel therapeutic drugs. The ocean, accounting for approximately 71% of the earth’s surface area, represents more than half of the biodiversity worldwide. Marine animals are important providers of natural active peptides (Chakniramol et al., 2022). At present, many bioactive peptides with anticancer potential have been identified from various marine animals (Figure 1), mainly concentrated in sponges, mollusks, marine arthropods, ascidians and fish (Zhang et al., 2021; Chakniramol et al., 2022; Wong et al., 2023). Some known ACPs from terrestrial animals (Figure 1), including terrestrial arthropods, amphibians, reptiles and mammals, have also been proven to have broad application prospects, serving as novel anti-tumor drugs or auxiliary therapeutic measures for cancer treatment (Pan et al., 2020; Tornesello et al., 2020). In Tables 1, 2, we comprehensively classified the natural ACPs of marine and terrestrial animals based on their order of evolution to the greatest extent possible, and provided a detailed introduction to the representative ACPs among them in the following section.

**TABLE 1:** Antimicrobial peptides (AMPs) with potential anticancer activity derived from marine animals, their application and mechanisms of action.

Peptides	Source	Group	Structure	Application	Mechanism of action	References
Jaspamide	*Jaspis johnstoni*	Sponges	Cyclic	Human prostate cancer, breast carcinomas, acute myeloid leukemia	Regulate caspase-3 protein, regulate the reduction of Bcl-2	Schweikart et al., 2013
Koshikamide H	*Theonella* sp.	Sponges	Cyclic	Human colon cancer	NR	Kang et al., 2018
Theonellamide G	*Theonella swinhoei*	Sponges	Cyclic	Human colon cancer	NR	Macedo et al., 2021
Callyaerins A–F and H	*Callyspongia aerizusa*	Sponges	Cyclic	Mouse lymphoma, human cervix carcinoma, rat brain tumor	NR	Ibrahim et al., 2010
Dolastatin 10	*Dolabella Auricularia*	Mollusks	Cyclic	Human lymphoma, lung cancer, ovarian carcinoma, prostate cancer, soft tissue sarcoma, breast cancer and pancreatic cancer	Inhibit microtubule assembly and tubulin polymerization, induce apoptosis and Bcl-2 phosphorylation	Gao et al., 2021
Kahalalide F	*Elysia rufescens*	Mollusks	Cyclic	Ovaries, breast, prostate, colon, liver tumor cells	Disturbance of lysosomal membrane, mediation of oncosis, alteration of cell membrane permeability and regulation of apoptosis	Wyer et al., 2022
KLH	*Megathura crenulata*	Mollusks	α-helical/β-sheet mixed	Breast, pancreatic, prostate cancer, esophageal adenocarcinoma	Regulation of apoptosis	Petrova et al., 2023
SALF	*Penaeus monodon*	Arthropods	Cyclic	Human cervical cancer	Regulate apoptosis related death receptor/NF-κB signaling pathway	Lin et al., 2010
Scyreprocin	*Scylla paramamosain*	Arthropods	α-helical	Human lung cancer	Accumulation of ROS and the release of Ca^2+^, dysfunction of mitochondrial function, activation of caspase-3	Yang et al., 2022
Ss-arasin	*Scylla serrata*	Arthropods	Random coil	Human cervical cancer, human colon carcinoma	NR	Anju et al., 2019
Penaeidin	*P. vannamei*	Arthropods	α-helical and random coil	Human kidney cancer	Induction of apoptosis	Meng et al., 2014
Tachyplesin I	*Tachypleus tridentatus*	Arthropods	β-sheet	Liver cancer, leukemia, gastric adenocarcinoma	Mediate the classic complement pathway	Li et al., 2003; Chen et al., 2005
Polyphemusin II	*Limulus polyphemus*	Arthropods	β-sheet	Human erythroleukemia	Induce necrotic cell death	Paredes-Gamero et al., 2012
Polyphemusin III	*Limulus polyphemus*	Arthropods	β-sheet	Human leukemia	Induce permeabilization of the cytoplasmic membrane	Marggraf et al., 2018
Styelin D	*Styela clava*	Ascidians	α-helical	Human cervical cancer	NR	Taylor et al., 2000
Turgencin A, B	*Synoicum turgens*	Ascidians	α-helical and random coil	Human melanoma cancer	NR	Hansen et al., 2020
Pardaxin	*Pardachirus marmoratus*	Fish	α-helical	Fibrosarcoma, oral squamous carcinoma, ovarian cancer, cervical carcinoma	Cell cycle arrest, induction of apoptosis, necrosis and autophagy	Wu et al., 2012; Huang and Chen, 2013; Lin et al., 2013; Han et al., 2015; Chen et al., 2021; Eghtedari et al., 2021
Piscidin-1	*Morone saxatilis* x *M.* *chrysops*	Fish	α-helical	Human fibrosarcoma	Induce apoptotic and necrotic activity	Lin et al., 2012
Piscidin-4	*Oreochromis niloticus*	Fish	α-helical	NSCLC	Regulate necrotic pathway	Ting and Chen, 2018
Epinecidin-1	*Epinephelus coioides*	Fish	α-helical	Human lung cancer and glioblastoma	Regulate cell apoptosis and necrosis	Su et al., 2020; Yu et al., 2023
MSP-4	*Oreochromis niloticus*	Fish	α-helical	Liver cancer, cervical cancer, fibrosarcoma, osteosarcoma	Induce the apoptosis	Kuo et al., 2018
TH1-5, TH2-3	*Oreochromis mossambicus*	Fish	α-helical/β-sheet mixed	Human fibrosarcoma, human cervical cancer, human breast cancer	Induce cell lysis, down-regulate the c-Jun gene, induce an inflammatory response, induce the apoptosis	Eghtedari et al., 2021
TFD-100	*Gadus macrocephalus*	Fish	Unknown	Human prostate cancer	Inhibit adhesion of PC3 to endothelial cells through binding to galactin-3	Guha et al., 2013
Chrysophsin-1	*Chrysophrys major*	Fish	α-helical	Human fibrosarcoma, histiocytic lymphoma, cervical carcinoma	Membrane depolarizing lytic mechanism	Hsu et al., 2011
NRC-03, NRC-07	*Pseudopleuronectes americanus*	Fish	α-helical	Multiple myeloma, breast carcinoma	DNA breakage, the accumulation of ROS	Hilchie et al., 2011, 2013
YALRAH	*Setipinna taty*	Fish	Linear	Prostate cancer	Induction of apoptosis	Song et al., 2014

NR, not reported.

**TABLE 2:** Antimicrobial peptides (AMPs) with potential anticancer activity derived from terrestrial animals, their application and mechanisms of action.

Peptides	Source	Group	Structure	Application	Mechanism of action	References
Melittin	*Apis mellifera*	Arthropods	α-helical	Human prostate cancer, ovarian carcinoma, glioma, astrocytoma, hepatocellular carcinoma, leukemic, lung tumor, squamous carcinoma, osteosarcoma and renal cancer	Induce apoptosis, cell cycle arrest and angiogenesis	Xu et al., 2017; Soliman et al., 2019; Yu et al., 2020
Mastoparan	*Polybia paulista*	Arthropods	α-helical	Human glioma, human bladder cancer, human lung cancer	Induction of apoptosis, alteration of mitochondrial permeability	Alhakamy et al., 2021
Decoralin	*Oreumenes decorates*	Arthropods	α-helical	Human breast cancer	Mediate the necrosis	Torres et al., 2018
Cecropins	*Hyalophora cecropia*	Arthropods	α-helical	Human leukemia, gastric carcinoma, bladder, kidney cancer, fibrosarcoma	Induction of necrosis	Qin et al., 2019
Harmoniasin	*Harmonia axyridis*	Arthropods	Unknown	Human leukemia	Induce apoptotic and necrotic cell deaths	Kim et al., 2013
Alloferon-1	*Calliphora vicina*	Arthropods	Unknown	Murine leukemia	Induce the cytotoxicity of natural killer cell	Chernysh et al., 2012
Smp24	*Scorpio maurus palmatus*	Arthropods	α-helical	Human leukemia, human lung cancer	Mediation of F-actin, regulation of MMP-2/9 and TIMP-1/2	Guo et al., 2022
AaeAP1a, AaeAP2a	*Androctonus aeneas*	Arthropods	α-helical, β-sheet and random coil	Human lung cancer, human prostate cancer, human breast cancer	NR	Du et al., 2015
Gomesin	*Acanthoscurria gomesiana*	Arthropods	β-sheet	Human melanoma and chronic myeloid leukemia	Target and destroy tumor cell membranes	Troeira Henriques et al., 2017
Lycosin-1	*Lycosa singoriensis*	Arthropods	α-helical	Human cervical, lung, prostate, colon and liver cancer, human fibrosarcoma	Inactivate STAT3 pathway, induce apoptosis	Shen et al., 2018
Ltc2a	*Lachesana tarabaevi*	Arthropods	α-helical	Human erythroleukemia	Target cancer cell membranes and form pores	Vorontsova et al., 2011
Scolopendrasin VII	*Scolopendra subspinipes mutilans*	Arthropods	Unknown	Human leukemia	Induction of necrosis	Lee et al., 2015
Scolopin-2	*Scolopendra subspinipes mutilans*	Arthropods	α-helical	Human cervical cancer, human liver cancer, human leukemia	Regulate caspase-related apoptosis pathways	Yan et al., 2018
MG2	*Xenopus laevis*	Amphibians	α-helical	Human lung cancer, human bladder cancer	Form pores on the cell membrane	Ghaly et al., 2023
Aurein 1.2	*Litoria aurea*	Amphibians	α-helical	Leukemia, melanoma, Lung, colon, ovarian, renal, prostate and breast cancer	Interact with cancer cell membranes	Rozek et al., 2000; Dennison et al., 2007
Hymenochirin-1B	*Hymenochirus boettgeri*	Amphibians	α-helical	Human lung cancer	Induce apoptosis and cell cycle arrest	Zhang et al., 2019
Dermaseptins	*Phyllomedusa bicolor*	Amphibians	α-helical	Human prostate cancer, breast cancer, B-lymphoma, glioblastoma, pancreatic cancer, melanoma	Interact with the lipids of the plasma membrane	Dos Santos et al., 2017
Alyteserin-2a	*Alytes obstetricans*	Amphibians	α-helical	Human breast, colon, lung and liver cancer	Inhibit the release of IL-10 and TGF-β	Conlon et al., 2013
BR-2R	*Rana ridibunda*	Amphibians	α-helical	Human breast cancer, human lung cancer	Induce apoptosis and cell cycle arrest	Hassanvand Jamadi et al., 2017
Kassinatuerin-3	*Kassina senegalensis*	Amphibians	α-helical	Human lung, prostate cancer and glioblastoma	NR	Wang et al., 2020
Crotamine	*Crotalus durissus terrificus*	Reptiles	α-helical/β-sheet mixed	Human pancreatic carcinoma, human melanoma, murine melanoma	Permeabilization of lysosomes, release of Ca^2+^ from endoplasmic reticulum, destruction of mitochondrial membrane potential	Urra and Araya-Maturana, 2022
BF-30	*Bungarus fasciatus*	Reptiles	α-helical	Mouse melanoma	Cytoplasmic membrane permeabilization and DNA-binding	Wang et al., 2013; Qi et al., 2020
KT2, RT2	*Crocodylus siamensis*	Reptiles	α-helical	Human colon cancer, human cervical cancer	Induction of apoptosis	Maijaroen et al., 2018
LL-37	*Homo sapiens*	Mammals	α-helical	Gastric cancer, human T leukemia, human oral squamous cell carcinoma, human colon cancer, human lung cancer	Activate tumor-suppressing bone morphogenetic protein, regulate caspase-independent apoptosis	Okumura et al., 2004; Mader et al., 2009; Wu et al., 2010; Ren et al., 2013; Yang et al., 2021
Defensins	*Homo sapiens*	Mammals	β-sheet	Human myeloid leukemia, lymphoblastoid B cells, cervical neoplasia	Membrane lysis, regulation of apoptosis, inhibition of neovascularization	Tornesello et al., 2020
Lactoferrin	*Bos taurus*	Mammals	β-sheet	Human leukemia, human neuroblastoma, human breast, colon, gastric and ovary cancer	Cell cycle arrest, induce cell apoptosis and necrosis, inhibit cell metastasis and angiogenesis, immune regulation	Rahman et al., 2021
ChMAP-28	*Capra hircus*	Mammals	α-helical	Human leukemia, human bosom adenocarcinoma, human epidermoid carcinoma, murine melanoma	Cause cell membrane permeability, induce necrotic death	Emelianova et al., 2018

NR, not reported.

### 3.1 ACPs isolated from marine animals

#### 3.1.1 ACPs derived from sponges

Sponges are filter-feeding animals that cope with hazardous particles by producing neutralizing bioactive compounds such as peptides. In the past 20 years, researchers have focused on sponges-isolated peptides such as jaspamide, koshikamides, and theonellamide G, which exhibited extensive cytotoxicity to various cancer cells. These peptides with unusual amino acids or non-amino acid parts compared to other animal derived ACPs. Jaspamide and koshikamides were the special structured cyclic depsipeptides found in the genus *Jaspis johnstoni* and the *Theonella* sp. respectively, exhibiting cytotoxicity to various cancer cells, like prostate, breast carcinomas, acute myeloid leukemia and colon cancer cells (Schweikart et al., 2013; Kang et al., 2018). Whereas, theonellamide G is a glycopeptide, obtained from the *Theonella swinhoei*, exhibiting cytotoxic effects against HCT-16 human colon adenocarcinoma cell line (Macedo et al., 2021).

#### 3.1.2 ACPs derived from marine mollusks

Marine mollusks rely on their physical barriers and innate immune systems to resist the most diverse pathogens, due to the adaptive immunity has not yet evolved. Since the 1990s, a few marine mollusks ACPs have been identified. Dolastatin 10 was the first marine mollusk ACP identified from the Indian Ocean sea hare *Dolabella Auricularia* (Bai et al., 1990), and is still considered one of the most powerful anti-cancer peptides found so far. It could inhibit the proliferation of human lymphoma, lung cancer, ovarian carcinoma, prostate cancer, soft tissue sarcoma, breast cancer and pancreatic cancer by inhibiting microtubule assembly and tubulin polymerization, and inducing apoptosis and Bcl-2 phosphorylation (Gao et al., 2021). Adcetris^®^ is an ADC drug modified from dolastatin 10, containing a CD30-specific mAb conjugated to monomethyl auristatin E (MMAE). It was approved by FDA in 2011 and became the second ADC drug to enter the oncology market (Tong et al., 2021).

#### 3.1.3 ACPs derived from marine arthropods

Arthropods are one of the most successful animals in adapting and surviving in the marine environment, not only due to their solid shells but also because of their complete inner immunity compared to other marine animals. AMPs play a vital in the immune system. Currently, various AMPs that have been proven as ACPs in marine arthropods are mainly concentrated in shrimps and crabs. For example, shrimp anti-lipopolysaccharide factor (SALF) displayed antitumor activity against Hela cells by apoptosis related death receptor/NF-κB signaling pathway. When combined with cisplatin, the inhibitory effect on Hela cells is more pronounced (Lin et al., 2010). The novel ACPs polyphemusins from the horseshoe crab *Limulus polyphemus* were discovered to against human cancer cells. In an anti-tumor research, polyphemusin II was examined to destroy the cell membranes of the human erythroleukemia K562 cell line by inducing necrotic cell death (Paredes-Gamero et al., 2012). Another study indicated that polyphemusin III could lead to rapid permeabilization of the cytoplasmic membrane of human leukemia cells HL-60 (Marggraf et al., 2018).

#### 3.1.4 ACPs derived from marine ascidians

Ascidians belong to the phylum of Chordata and are the most evolved group of marine organisms. Unique cyclic and linear peptides containing unusual amino acids derived from ascidians have increased our knowledge regarding novel ACPs. Styelin D, an ACP with 32 residues containing two specific amino acids (dihydroxyarginine and dihydroxylysine) from blood cells of the ascidian *Styela clava*, showed significant cytotoxic and hemolytic to human cervical cancer epithelial cell line ME-180 (Taylor et al., 2000). Moreover, turgencin A and turgencin B with six cysteine residues are two novel linear ACPs, derived from the ascidian *Synoicum turgens*, which showed obvious anticancer activities to suppress the proliferation of melanoma cancer cell line A2058 and the human fibroblast cell line MRC-5 (Hansen et al., 2020).

#### 3.1.5 ACPs derived from fish

Bioactive peptides are indispensable components of fish innate immune system, to prevent the invasion of pathogens from the aquatic microorganisms ecosystems. Fish ACPs mainly identified from the fish mucus, among them, pardaxin, piscidin, and epinecidin-1 are the representative beings. Pardaxin is the most widely studied ACP present in the Red Sea Moses sole (*Pardachirus marmoratus*). It is composed of α-helix with 33 amino acid residues and has been experimentally confirmed in several studies on different cancer cell lines to show anticancer potential through multiple mechanisms of action. For example, pardaxin could arrest the cell cycle of cancer cells in the G2/M phase, thereby interfering with the cell proliferation process (Eghtedari et al., 2021). Moreover, pardaxin has been researched for its antitumor properties via inducing apoptotic cell death in various cancer cell lines such as fibrosarcoma (Wu et al., 2012), oral squamous carcinoma (Han et al., 2015), ovarian cancer cells (Chen et al., 2021) and cervical carcinoma (Huang and Chen, 2013). Besides that, pardaxin was also demonstrated to cause cell necrosis in fibrosarcoma (Lin et al., 2013) and autophagy in ovarian cancer cells (Chen et al., 2021). Furthermore, it is particularly noteworthy that the endoplasmic reticulum (ER)-targeting ability of pardaxin in cancer cells (Qin et al., 2020). The piscidin family is an important component of the innate immunity of teleost, and recent studies have shown that they have extensive antibacterial and anticancer activities. A study reported that piscidin-1 could inhibit the motility and proliferation of HT1080 (human fibrosarcoma cell line) via inducing apoptotic and necrotic activity (Lin et al., 2012). Another study observed that piscidin-4 displayed significant cytotoxicity toward non-small cell lung cancer (NSCLC) cell lines such as A549, NCI-H661, NCI-H1975 and HCC827 (Ting and Chen, 2018). In this research, piscidin-4 caused NSCLC cell death through the necrotic pathway rather than the apoptotic pathway. Another common peptide was epinecidin-1, a naturally occurring peptide isolated from orange-spotted grouper (*Epinephelus coioides*), which exhibited inhibitory activity on human lung cancer and glioblastoma by regulating the process of cell apoptosis and necrosis (Su et al., 2020; Yu et al., 2023).

### 3.2 ACPs isolated from terrestrial animals

#### 3.2.1 ACPs derived from terrestrial arthropods

Terrestrial arthropods are rich sources of novel bioactive compounds, including ACPs, which are mainly derived from three main classes: Insecta, Arachnida, and Chilopoda.

Insect AMPs are critical factors of the insect immune system, and also show toxic effects on many cancer cells (Table 2). Bee venom is a kind of important insect-derived AMPs with anti-tumor effects. Melittin is an amphiphilic bioactive peptide derived from the honey bee *Apis mellifera* and is the most researched and representative bee venom-derived AMP. Melittin induced a variety of cell death mechanisms, such as apoptosis, cell cycle arrest, and angiogenesis (Soliman et al., 2019). Experimental studies have proven that melittin exhibited anticancer activity in prostate cancer, ovarian carcinoma, glioma, astrocytoma, hepatocellular carcinoma, leukemic, lung tumor, squamous carcinoma, osteosarcoma and renal cancer cells (Xu et al., 2017; Soliman et al., 2019; Yu et al., 2020). However, the use and application of melittin in clinical experiments was limited because of its widespread cytotoxicity against both tumor cells and normal cells. Therefore, the recent research hotspots of melittin mainly focused on developing safe and stable melittin delivery systems (Wang A. et al., 2022). In addition, A classic AMP from silkworms is cecropin, which was considered as a necrosis-inducing peptide and exhibited anti-proliferation activity to leukemia cells, gastric carcinoma, bladder cancer, fibrosarcoma and HCC (Qin et al., 2019).

The ACPs in Arachnida are mainly concentrated in the venom of scorpions and spiders (Table 2). Smp24, a cationic AMP isolated from the venom gland of the Egyptian scorpion *Scorpio maurus palmatus*, has been proven to show potent suppressing effects on leukemic tumor cell lines (KG1-a and CCRF-CEM) and human lung cancer cell lines (A549, H3122, PC-9, and H460). Furthermore, its inhibition of the A549 cell migration ability is through the mediation of filamentous actin (F-actin) and the changes of MMP-2/9 and TIMP-1/2 protein expressions (Guo et al., 2022). Spider AMPs with anti-cancer activities have also been reviewed. Spider venom-derived peptide lycosin-1 was proven to inhibit seven cancer cell lines proliferation *in vitro* and effectively suppressed tumor growth *in vivo*. Mechanistically, it mediated the mitochondrial death pathway, making cancer cells sensitive to apoptosis, and inactivated the STAT3 pathway (Shen et al., 2018).

Centipedes are typical venomous arthropods. Lee et al. (2015) reported an ACP isolated from the Centipede, *Scolopendra subspinipes mutilans* called scolopendrasin VII. The results showed that scolopendrasin VII reduced the viability of the leukemia cells by inducing necrosis. Scolopin-2, a cationic AMP from centipede venoms, displayed anti-proliferation activities to tumor cell lines such as HeLa, HepG2 and K562 and did not show toxic side effects on normal cells (Yan et al., 2018).

#### 3.2.2 ACPs derived from amphibians

The skin secretions of amphibians are composed of various bioactive peptides, and hundreds of skin AMPs have been isolated from frogs and toads (Oelkrug et al., 2015), some of which also exhibited selective cytotoxicity against cancer cells (Conlon et al., 2014). For example, MG2, isolated from *Xenopus laevis* skin, has demonstrated antitumor activity against human lung cancer and bladder cancer via forming pores on the cell membrane (Ghaly et al., 2023). Aurein 1.2 is a peptide found in the frog *Litoria aurea*, which was reported to exhibit high activity against 55 different cancer cell lines *in vitro* (Rozek et al., 2000; Dennison et al., 2007). Furthermore, hymenochirin-1B is a cationic α-helix peptide belonging to the hymenochirin family from the frog *Hymenochirus boettgeri*, and has been reported to exert antineoplastic activities against lung cancer cells NCI-H1299 and A549 by inducing apoptosis and cell cycle arrest through the mitochondrial pathway (Zhang et al., 2019). Additionally, many other ACPs from amphibians and their mechanisms of action are summarized in Table 2.

#### 3.2.3 ACPs derived from reptiles

Reptile-derived ACPs mainly from the venom of snakes. Crotamine is one of the main peptides of the venom of the South American rattlesnake *Crotalus durissus terrificus*. It acted on the permeabilization of lysosomes, the release of Ca^2+^ from the endoplasmic reticulum and the destruction of mitochondrial membrane potential (Urra and Araya-Maturana, 2022), then showed cytotoxicity toward various cancer cells, such as human pancreatic carcinoma cells Mia PaCa-2, human melanoma cells SK-Mel-28 and murine melanoma cells B16-F10. Another remarkable ACP containing 30 amino acids named cathelicidin-BF (BF-30) was found in the snake *Bungarus fasciatus*. In a study, BF-30 suppressed the growth of mouse melanoma cell B16F10 in a dose and time-dependent manner. *In vivo* experiments, BF-30 obviously inhibited melanoma development in B16F10 tumor-bearing mice without adverse reactions occurring (Wang et al., 2013). In addition, Qi et al. (2020) designed a BF-30 derivative, LBF-14, which exhibited significant anti-melanoma action through disrupting the cell membrane, and then binding to the genomic DNA to suppress the migration and angiogenesis of melanoma cells.

#### 3.2.4 ACPs derived from mammals

Several representative ACPs in mammals have been extensively studied (Table 2). Among them, three of the peptides that have been extensively studied are LL-37, defensins, and lactoferricin. The human cathelicidin, LL-37, participated in the killing process of various tumor cells. For example, Wu et al. (2010) indicated that LL-37 showed cytotoxicity to gastric cancer cells by activating tumor-suppressing bone morphogenetic protein signaling via suppression of proteasome. LL-37 could also inhibit the growth of Jurkat human T leukemia cells, human oral squamous cell carcinoma (OSCC) cells SAS-H1 and colon cancer cells via caspase-independent apoptosis (Okumura et al., 2004; Mader et al., 2009; Ren et al., 2013). Importantly, LL-37 has been modified to many shorter sequences to exhibit lower toxicity and higher efficiency. 17BIPHE2, the shortest modified LL-37, could induce apoptosis of human lung cancer better than LL-37 (Yang et al., 2021). Defensins, an important group of cysteine-rich and β-sheet AMPs widely presented in various mammals, have been discovered to display cytolytic activity against several cancer cell lines, such as human myeloid leukemia cell line U937, K562, lymphoblastoid B cells IM-9 and WIL-2 and cervical neoplasia. Furthermore, the anti-cancer activities of α-defensins, including the human neutrophil peptides (HNP) 1–3, have been proven through the manner of membrane lysis and apoptosis as well as inhibition of neovascularization in tumor cells (Tornesello et al., 2020). Lactoferricin is a cationic peptide identified from the acid-pepsin hydrolysis of lactoferrin in mammalian milk and exhibited cytotoxic activity against a panel of microorganisms and cancer cells. Lactoferricin mainly exerted anti-tumor effects through cell cycle arrest, inducing cell apoptosis and necrosis, inhibiting cancer cell metastasis and angiogenesis, and immune regulation (Rahman et al., 2021).

## 4 Mechanisms of AMPs underlying their anti-cancer effects

Due to the non-specific destruction of the plasma membrane by ACPs, they exhibit therapeutic potential for tumors that are ineffective in traditional drug therapy. Although some of the main mechanisms of action have been reported, the exact mechanisms of the toxic effect of ACPs on cancer cells remain controversial. Generally speaking, the anti-cancer effect of bioactive peptides can be achieved by regulating membrane or non-membrane mechanisms (Harris et al., 2013).

### 4.1 Membranes disruptive mechanisms

**FIGURE 2:**
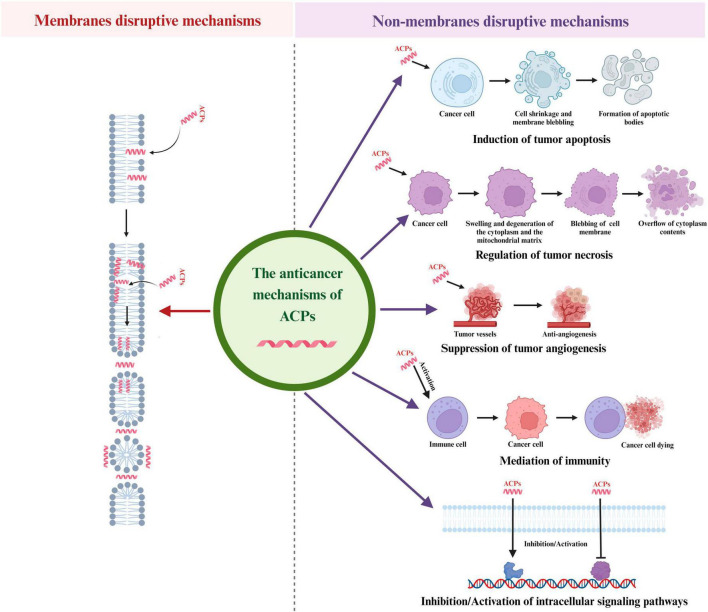
Different anticancer mechanisms of ACPs. ACPs can function through a variety of mechanisms, including induction of tumor apoptosis, regulation of tumor necrosis, suppression of tumor angiogenesis, mediation of immunity and regulation of intracellular signaling pathways.

As mentioned in the section “2 The structural classifications and selective recognition to tumor cells of AMPs” of this article, the destruction modes of ACPs on tumor cell membranes is similar to that of AMPs induced bacterial membrane damage (Figure 2). For example, a study reported by Papo et al. (2006) indicated that a short host defense-like peptide could selectively recognize cancer cells mainly via binding with negatively charged PS on the cell surface, leading to depolarization of cell plasma membrane and cell death. Therefore, peptide-lipid interaction is the committed step to effectively destroy tumor cell membranes. Another research also demonstrated that the spider venom Ltc2a could induce tumor cell membrane blebbing, swelling, and ultimately cell death. Interestingly, this peptide interacted with the outer membrane leaflet of tumor cells, thereby inducing PS externalization. Due to the formation of membrane pores in tumor cells, their permeability to anionic molecules is stronger than that of cationic molecules, and the redistribution of PS toward the outer leaflet of the membrane was discovered in the tumor cells (Vorontsova et al., 2011). Moreover, several ACPs such as cecropins, pardaxin, magainins and melittin were proven that they induce the formation of pores in tumor cell membranes through the barrel-stave model, toroidal model and carpet model, and lead to tumor lysis and death (Ryan et al., 2013; Han et al., 2015; Kashiwada et al., 2016; Pino-Angeles et al., 2016; Zhang S. K. et al., 2016).

### 4.2 Non-membranes disruptive mechanisms

#### 4.2.1 Induction of tumor apoptosis

Apoptosis is a gene-directed program developed by multicellular organisms to control cell proliferation to cope with DNA damage during development or after cell stress. The characteristics of the apoptosis process are cell shrinkage, membrane blebbing, chromatin condensation and nuclear fragmentation and then small membrane-bound apoptotic bodies are released and swallowed by macrophages or adjacent cells. There are two major pathways of apoptosis in mammalian cells: mitochondrial-mediated endogenous pathways and death receptor-mediated exogenous pathways. In the mitochondrial-mediated endogenous pathways of apoptosis, cell apoptosis is caused by mitochondrial dysfunction and membrane damage, resulting the release of the Cytc. The Cytc-induced apoptosis is mainly initiated by the apoptotic protease (Caspase) pathway (Dave et al., 2011). The early opening of the mitochondrial permeability transition pore (mPTP) in the inner mitochondrial membrane (IMM) is a key reason in primary necrosis. These changes lead to extreme swelling of mitochondrial permeability, ultimately resulting in necrotic cell death (Mulder et al., 2013). In the death receptor pathway, various external factors act as promoters of cell apoptosis, and then transmit apoptotic signals through different signal transduction systems, inducing cell apoptosis. Fas and Fas ligand (FasL/CD95L) are the two most important apoptosis-inducing molecules in cancer cells (Shimoyama et al., 2015). Cell apoptosis plays a vital role in eliminating cancer cells without causing damage to normal cells or surrounding tissues. Regulating the apoptosis pathway in tumor cells will be an effective method for cancer prevention and treatment, and peptides that can induce tumor cell apoptosis (Figure 2) are gradually becoming significant candidates for the development of novel anticancer medicines. Ceremuga et al. (2020) found a dose dependent decrease of mitochondrial membrane potential in human leukemia cells treated with melittin, which confirmed the process of cell apoptosis and suggested a potential pathway related to the intrinsic mechanism that depended on mitochondria. A study in 2022 demonstrated that NRC-03 could induce apoptosis in OSCC cells through the cyclophilin D (CypD)-mPTP axis mediated mitochondrial oxidative stress (Hou et al., 2022). In terms of exogenous pathways mediated by death receptors, SALF is a typical peptide that exerted anti-tumor effects through the apoptosis-related death receptor/NF-κB signaling pathway (Lin et al., 2010). Moreover, in recent years, researchers have also found that both endogenous and exogenous apoptosis pathways were involved in ACPs induced cancer cell apoptosis. For example, MSP-4 could induce apoptosis via activation of extrinsic Fas/FasL- and intrinsic mitochondria-mediated pathways in the osteosarcoma cell line (Kuo et al., 2018). In another study, the expression of the cell apoptosis key proteins, such as Cytc, caspase-9, and caspase-3 in the endogenous mitochondrial pathway, and Fas, FasL in the exogenous death receptor pathway were significantly up-regulated after treating lung cancer cells with dermaseptin (Dong et al., 2020). In addition, Ju and co-workers found that both the caspases mediating extrinsic and mitochondria intrinsic pathways were activated in Brevinin-1RL1 induced apoptosis (Ju et al., 2021).

#### 4.2.2 Regulation of tumor necrosis

Necrosis is defined as an unexpected, uncontrolled, and non-programmed form of cell death. Accidental necrosis is usually caused by chemical or physical damage, ultimately leading to chromatin flocculation, swelling and degeneration of the cytoplasm and the mitochondrial matrix, blebbing of the cellular membrane, and overflow of cytoplasm contents to the outside of cells (Padanilam, 2003). Necrosis inducing peptides are a group of lytic peptides that destroy cell membranes (Figure 2). Compared with traditional chemotherapy drugs, they have higher selectivity toward cancer cells and do not induce multidrug resistance, making them a promising new class of anticancer drugs (Bhutia and Maiti, 2008). For instance, Su et al. (2019) found that piscidin 4 displayed anti-tumor activity on glioblastoma cell lines via inducing mitochondrial hyperpolarization and mitochondrial dysfunction, followed by DNA damage and necrosis. A study in 2019 revealed that melittin induced cell death mostly occurred by necrosis in MCF-7 and MDA-MB-231 cells (Daniluk et al., 2019). Moreover, it was reported that decoralin could induce necrosis in cancer cells by enhancing the stiffness of the cell membrane (Mirzaei et al., 2021). It was also pointed out that cathelicidin reduced the tumor growth and regulated necrosis of cancer cells by releasing tumor necrosis factor-alpha (TNF-α) and granules enzymes (Mahmoud et al., 2022). Furthermore, Smp24 could induce necrosis of A549 cells through damaging the integrity of the cell membrane, mitochondrial and nuclear membranes (Guo et al., 2022).

#### 4.2.3 Suppression of tumor angiogenesis

Angiogenesis is the process through which novel blood vessels are formed from pre-existing ones and it is a multi-step process involving endothelial cell (EC) proliferation and migration (Starzec et al., 2006). Angiogenesis plays an important role in the growth, invasion, and metastasis of tumors. Specifically, nutrition, oxygen supply, and metabolic waste excretion in tumors are regulated through a complex network of tumor microvessels (De Palma et al., 2017). Tumor angiogenesis is completed via a range of sequential steps, which further result in the development of cancer. The process of angiogenesis is mainly triggered by the tumor itself, and as malignant tumors continue to grow, tumor cells become hypoxic (Marme, 2018). Hypoxia is the basic initiating factor for tumor angiogenesis (De Palma et al., 2017). Moreover, there are many growth factors involved in tumor angiogenesis, such as vascular endothelial growth factor (VEGF), epidermal growth factor (EGF), angiogenin (Ang), fibroblast growth factor (FGF), platelet-derived growth factor (PDGF), TNF-α and placental growth factor (PLGF) (Al-Ostoot et al., 2021). Therefore, inhibiting angiogenesis is an effective way to hinder tumor development. Many bioactive peptides exert effective anti-angiogenic and anti-cancer effects mainly by blocking the interaction between growth factors and their receptors (Figure 2). For example, Zhang Z. et al. (2016) pointed out that melittin was capable of suppressing cathepsin S-induced angiogenesis by blocking of the VEGF-A/VEGFR-2/mitogen-activated protein kinase 1 (MEK1)/extracellular signal-regulated kinase 1/2 (Erk1/2) signaling pathway. Moreover, it has been proved that lactoferrin could reduce the growth of blood vessels in colon cancer cells and the mechanism was validated to be associated with the inhibition of angiogenesis related pathways, including VEGFR2, vascular endothelial growth factor A (VEGFA), PI3K, Akt, and Erk1/2 (Li et al., 2017). Another study indicated that the downregulation of FGFR1 was an indication of the novel anti-angiogenic activity of dermaseptins in rhabdomyosarcoma cells (Abdille et al., 2021).

#### 4.2.4 Mediation of immunity

Malignant tumor cells recruit multiple cell types through generating cytokines and growth factors, such as endothelial cells, inflammatory immune cells, and fibroblasts (Liotta and Kohn, 2001). These different types of cells and extracellular matrix molecules constitute the tumor microenvironment (Devaud et al., 2013). Inflammatory reactions are involved in various biological processes (Medzhitov, 2008; Wang P. et al., 2022). In the tumor microenvironment, there is a delicate balance between anti-tumor immunity and tumor-derived proinflammatory activity, which limits the progress of anti-tumor immunity (Murugaiyan and Saha, 2013). Many peptides display cytotoxicity against tumor cells by activating the human immune system, thereby achieving the goal of curing cancer (Figure 2). Research has shown that both alloferon-1 and alloferon-2 could induce the cytotoxicity of natural killer cells in mammals, such as mice and humans. In a mouse tumor transplantation model, alloferon-1 monotherapy showed moderate tumor suppression comparable to low-dose chemotherapy (Chernysh et al., 2012). Another study indicated that lactoferrin could up-regulate the components of non-specific immunity against cancer, including interferon-γ (IFN-γ), caspase-1 and interleukin-18 (IL-18). When interacting with danger associated molecules (DAMPs) derived from cancer cells, membrane components of innate immunity, such as pattern recognition receptor (PRR), trigger a response of inflammasomes, resulting in activation of caspase-1, which cleaves pro-IL18 and produces IL-18. IL-18 is a potent pro-inflammatory cytokine that acts as an IFN-γ-inducer factor in NK and T cells with effector cell lysis activity on cancer cells (Cui et al., 2014). Additionally, Tang et al. (2022) designed a tumor microenvironment (TME) responsive MnO_2_-melittin nanoparticles (M-M NPs). The M-M NPs could activate cGAS-STING pathway and promote the maturation of antigen-presenting cells to initiate systemic anti-tumor immune response such as increasing the production of tumor-specific T cells and more pro-inflammatory cytokines and chemokines.

#### 4.2.5 Regulation of other intracellular signaling pathways

A more possible mechanism of cancer cell growth inhibitory activity of AMPs is associated with modulating various cell regulatory/signaling pathways intracellularly (Figure 2). Janmaat and others have demonstrated that kahalalide F exerted cytotoxic activity on cancer cells by regulating ErbB3 and the downstream PI3K-Akt signaling pathway (Janmaat et al., 2005). Another study mentioned that melittin suppressed the PI3K/Akt/mTOR signaling pathway, leading to an inhibition of EGF-induced breast cancer cell migration and invasion (Jeong et al., 2014). A report of Uen et al. (2019) found that pardaxin exhibited anti-cancer activity to leukemic cells via regulating toll-like receptor (TLR2)/MyD88 signaling pathway. Specifically, padaxin induced leukemia cells to differentiate into macrophages like cells with immunostimulatory functions, including phagocytic ability and production of superoxide anions, by activating MyD88 protein. Furthermore, cecropin A has been shown to down-regulate MEK/ERK phosphorylation. The MAPK/ERK signaling pathway is responsible for the regulation of cell division, cell cycle or transcription regulation in cancer cells (Zhai et al., 2019). A recent study in 2022 confirmed that melittin could inhibit the growth of osteosarcoma 143B cells, which was associated with the supression of Wnt/β-catenin signaling pathway activity and cause apoptosis through up-regulating the ratio of Bax/Bcl-2 (Xie et al., 2022).

## 5 Clinical applications of ACPs

Although significant progress has been made in the treatment and prevention of various cancers, one of the main issues with cancer therapies is heavy side effects. In contrast, the ACP agents exhibit cytotoxicity against cancer cells at low concentrations without toxicity to normal tissues and therefore provide new strategies for cancer treatment. In addition, the peptides also have characteristics such as small size, simple synthesis, ability to enter the cell layer, high specificity and affinity, and lower immunogenicity than chemotherapy drugs (Eghtedari et al., 2021). At last, another advantage of using peptides as therapy is that they do not accumulate in the kidney or liver, which greatly limits their toxic reactions (Marqus et al., 2017).

Several peptide anti-tumor drugs have been completed or are currently undergoing clinical trials. For instance, balixafortide is based on polyphemusin, a natural peptide from the crab. The completed results of phase I and II trials have shown that the combination of balixafortide and ibuprofen enhanced the anti-tumor effect of HER2 negative metastatic breast cancer patients and improved tolerance compared to ibuprofen alone, without producing dose limiting toxicities (Pernas et al., 2018). The blockade of the C-X-C chemokine receptor type 4 (CXCR4) pathway by balixafortide played a crucial role in inhibiting metastasis and diffusion, making tumor cells sensitive to chemotherapy, and activating the immune system in the TME. Therefore, balixafortide may show great potential for its forthcoming phase III trial (NCT03786094). LL-37 is the most studied human ACP and the completed clinical trial (NCT02225366) in 2021 evaluated the appropriate dose of LL-37 for intratumoral administration in melanoma patients (Jafari et al., 2022). Another ACP called LTX-315, a lactoferrin-derived lytic peptide, is currently undergoing a phase I multicenter study (NCT01058616) to evaluate its therapeutic efficacy against various types of transdermal accessible tumors.

In the past few decades, the USFDA has approved several ACP drugs. For example, gonadotropin-releasing hormone (GnRH) analog leuprolide acetate (Lupron^®^) was used to treat prostate cancer in 1990. It possessed immunomodulatory effects including reduction of the production of TNF-α, IL-1β and other inflammatory cytokines to exert anti-cancer function (Guzmán-Soto et al., 2016). Adcetris^®^ is an ADC drug developed based on dolastatin 10 for the treatment of lymphoma, which was launched in 2011 and successfully overcame obstacles in the clinical application of dolastatin 10 as a single drug. Before Adcetris^®^ was developed, dolastatin 10 failed in phase II clinical trial owing to adverse reactions such as hematological and vascular toxicities (von Mehren et al., 2004). Subsequently, researchers used ADC technology to couple monoclonal antibodies with dolastatin 10 derivatives and utilized the specificity of antibodies to transport drugs to target tissues for their anti-cancer functions. At last, Adcetris^®^ effectively reduced the systemic toxicity and side effects.

## 6 Conclusion

Terrestrial and marine animals contain abundant bioactive peptides that have been successfully developed and have achieved good therapeutic effects, such as antioxidant, antibacterial, anticancer, immune regulation, etc. In this review, we summarized the main animal-origin ACPs according to the animal’s evolution order and their anti-tumor mechanisms that have been reported in the past decades. Comprehensively, ACPs exert anti-cancer activity through membrane disruptive and non-membrane disruptive mechanisms including mediation of the necrosis or apoptosis of cancer cells, inhibition of angiogenesis, recruitment of immune cells and activation of certain regulatory functional proteins. Then we reviewed the clinical applications of ACPs. Encouragingly, an increasing number of peptide anti-tumor drugs have been approved for commercialization by the USFDA or undergoing clinical trials. In all, we expect that, with the accumulation of ACPs conducted in clinical trials in the future, ACPs could become one of the novel choices for the clinical treatment of cancers.

## Author contributions

BQ: Conceptualization, Writing – original draft. JY: Conceptualization, Writing – original draft. XL: Methodology, Writing – original draft. SZ: Supervision, Visualization, Writing – review and editing. XM: Writing – review and editing. LL: Project administration, Writing – review and editing.

## Footnotes

1
https://aps.unmc.edu/
